# QuadNet: A Hybrid Framework for Quadrotor Dead Reckoning

**DOI:** 10.3390/s22041426

**Published:** 2022-02-13

**Authors:** Artur Shurin, Itzik Klein

**Affiliations:** The Hatter Department of Marine Technologies, University of Haifa, Haifa 3498838, Israel; ashurin@campus.haifa.ac.il

**Keywords:** drones, deep learning, inertial measurement unit, indoor navigation, quadrotor dead reckoning

## Abstract

Quadrotor usage is continuously increasing for both civilian and military applications such as surveillance, mapping, and deliveries. Commonly, quadrotors use an inertial navigation system combined with a global navigation satellite systems receiver for outdoor applications and a camera for indoor/outdoor applications. For various reasons, such as lighting conditions or satellite signal blocking, the quadrotor’s navigation solution depends only on the inertial navigation system solution. As a consequence, the navigation solution drifts in time due to errors and noises in the inertial sensor measurements. To handle such situations and bind the solution drift, the quadrotor dead reckoning (QDR) approach utilizes pedestrian dead reckoning principles. To that end, instead of flying the quadrotor in a straight line trajectory, it is flown in a periodic motion, in the vertical plane, to enable peak-to-peak (two local maximum points within the cycle) distance estimation. Although QDR manages to improve the pure inertial navigation solution, it has several shortcomings as it requires calibration before usage, provides only peak-to-peak distance, and does not provide the altitude of the quadrotor. To circumvent these issues, we propose QuadNet, a hybrid framework for quadrotor dead reckoning to estimate the quadrotor’s three-dimensional position vector at any user-defined time rate. As a hybrid approach, QuadNet uses both neural networks and model-based equations during its operation. QuadNet requires only the inertial sensor readings to provide the position vector. Experimental results with DJI’s Matrice 300 quadrotor are provided to show the benefits of using the proposed approach.

## 1. Introduction

In the last decade, quadrotor usage for both civilian and military applications has significantly increased in applications such as construction, transportation, surveillance, industry, marine science, mapping, military, emergency response, and law enforcement. In construction and industry, quadrotors are used to examine the condition of structures, machinery, or infrastructure located in remote areas or at high altitudes. When surveying areas that are difficult to access on wheels or foot, quadrotors easily perform that kind of task [1,2]. In transportation, quadrotors are used for data collection, patrol, and recently for deliveries [3,4]. Quadrotors are employed in marine science studies for marine animal research [5], marine species identification, and photogrammetric studies [6]. For mapping applications, quadrotors scan an area and take pictures of it to build a virtual 3D model [7], later used for mobile 3D mapping and image recognition. Quadrotors are also employed for grasping and retrieving objects while hovering, as seen in [8,9]. In emergency situations such as rescue missions, quadrotors may be used when foot or vehicular access is dangerous or difficult, for example, buildings after natural disasters or terror attacks [10].

To accomplish the challenging tasks assigned to them, quadrotors require an accurate navigation system. Using a global navigation satellite systems (GNSS) receiver combined with an inertial navigation system (INS) is one of the commonly used approaches for quadrotor navigation. It provides position and velocity information with an accuracy suitable for various applications [11,12]. However, indoors and some outdoor locations (like urban canyons), GNSS readings might not be available and the navigation solution relies only on the INS solution in a situation known as pure inertial navigation. There, due to noise and errors in the inertial sensors, the navigation solution drifts in time.

As GNSS measurements are not available indoors, quadrotor navigation is usually based on the fusion between the INS and a camera.

In [13,14,15] simultaneous localization and mapping (SLAM) approaches are used as an alternative to GNSS indoors. In [16], a quadrotor indoor navigation algorithm based on position-based visual servoing was suggested, while indoor quadrotor navigation based on visual markers for quadrotor position computation was used for library inventory and book localization was suggested in [17]. Both visual positioning systems and SLAM rely on a camera as a main sensor. However, in some situations, such as poor visibility, the camera is not usable. Therefore, as GNSS suffers from blockage and unavailability, and cameras from lighting conditions and distortion, the quadrotor navigation solution is expected to rely only on the inertial sensors in several portions of its trajectory. As a consequence, the navigation solution drifts with time [18].

**Figure 1:**
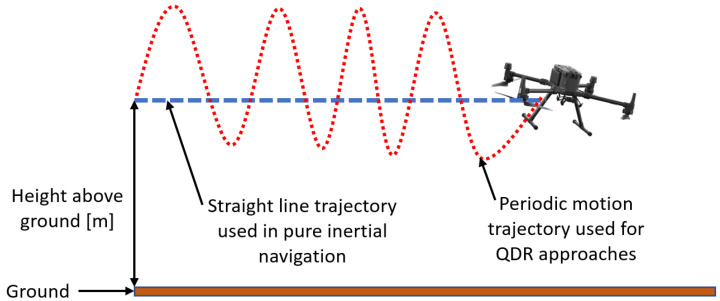
Illustration of a periodic motion used in the QDR approach.

Recently, to cope with situations of pure inertial navigation, the quadrotor dead-reckoning (QDR) framework was proposed [19]. Motivated by the pedestrian dead reckoning (PDR) approach using the smartphone inertial sensors [20,21,22], QDR requires the quadrotor to be flown in a periodic motion trajectory instead of a straight line trajectory. In this manner, similar to step-length detection and estimation in PDR, the peak-to-peak change in distance of the quadrotor is estimated. Figure 1 illustrates the straight line trajectory used in pure inertial navigation and also the periodic motion trajectory applied in the QDR approach.

QDR manages to significantly improve the pure inertial navigation solution; however, it has several shortcomings. QDR requires a gain calibration before it can be used and this gain is limited to the periodic motion applied in the process. In addition, QDR provides only a change in distance estimation between two peaks of the period motion along the trajectory, and so the ability to track the actual trajectory is limited to the time between those peaks, which could be several seconds. Finally, QDR provides only the change in distance of the quadrotor and does not provide its altitude; thus, it is limited to a two-dimensional position solution.

In other related navigation domains, machine learning (ML) and deep learning (DL) algorithms are used to improve the overall navigation performance. In [23,24,25], a deep learning approach is used for robot indoor navigation. Human activity recognition [26,27,28] and smartphone location recognition (SLR) [29] algorithms based on ML/DL were shown to improve the accuracy of PDR by using it as a prior [30,31]. SLR was also shown to improve the performance adaptive attitude and heading reference system (AHRS) [32]. ML approaches were also used to improve the accuracy and time to converge of the coarse alignment process [33]. In autonomous underwater vehicles, an end-to-end DL approach was suggested to regress missing Doppler velocity log measurements [34]. In addition, DL approaches greatly improved traditional PDR approaches [35,36,37,38,39].

In this paper, we propose QuadNet, a hybrid DL-framework for quadrotor dead reckoning enabling three-dimensional position estimation at any user-defined time rate using only inertial sensor readings.

QuadNet, being a hybrid framework, uses regression neural networks to provide the quadrotor’s change in distance and altitude and model-based equations to determine the heading. Thus, QuadNet requires only the inertial sensor readings to provide the three-dimensional position vector. Two different network architectures for the regression task are suggested and evaluated.

The main contribution of the paper is an accurate pure quadrotor inertial navigation solution. Compared to the model-based QDR solution [19], the proposed approach:

1. Provides altitude information enabling the determination of the quadrotor three-dimensional position vector;
2. The regression rate can be set at any desired time interval for the position vector estimation. For example, in [19], it was a peak-to-peak estimation occurring approximately every seven seconds, and now it was reduced to less than a second.
3. As a data-driven approach, there is no need for any calibration prior operation as required in the model-based QDR approach.

Experimental results with DJI’s Matrice 300 quadrotor are provided to show the benefits of using the proposed approach over the QDR approach in situations of pure inertial navigation.

The rest of the paper is organized as follows: Section 2 presents the INS equations of motion and the QDR approach. Section 3 presents the proposed QuadNet framework, and Section 4 elaborates on the data collection process and prepossessing. Section 5 describes the experiment results, and Section 6 provides the conclusions.

## 2. Problem Formulation

### 2.1. Inertial Navigation Systems

The specific force vector, ${\tilde{f}}_{ib}^{b}$, as measured by the accelerometers is:

(1) $${\tilde{f}}_{ib}^{b}=\left[\begin{array}{c} f_{x}\\ f_{y}\\ f_{z} \end{array}\right]$$

where $f_{x},f_{y}$, and $f_{z}$ are the vector components of the specific force vector as measured along the accelerometer-sensitive axes. In the same manner, the angular velocity vector, ${\tilde{\omega }}_{ib}^{b}$, as measured by the gyroscopes is:

(2) $${\tilde{\omega }}_{ib}^{b}=\left[\begin{array}{c} \omega_{x}\\ \omega_{y}\\ \omega_{z} \end{array}\right]$$

where $\omega_{x},\omega_{y}$, and $\omega_{z}$ are the vector components of the angular velocity vector as measured along the gyroscopes’ sensitive axes.

The inertial sensor readings (1)–(2) and initial conditions are used to solve the INS equations of motion to obtain the navigation solution: position, velocity, and attitude. The INS equations of motion expressed in the navigation frame, neglecting the angular rate of the Earth and Transport rate, are given by [18]:

(3) $$\begin{array}{ccc} {\dot{p}}^{n} & = & v^{n} \end{array}$$

(4) $$\begin{array}{ccc} {\dot{v}}^{n} & = & T_{b}^{n}{\tilde{f}}_{ib}^{b}+g^{n} \end{array}$$

(5) $$\begin{array}{ccc} {\dot{T}}_{b}^{n} & = & T_{b}^{n}\Omega_{ib}^{b} \end{array}$$

where $p^{n}$ is the position vector expressed in the local navigation frame, $v^{n}$ is the velocity vector expressed in the navigation frame, $g^{n}$ is the local gravity vector expressed in the navigation frame, $\Omega_{ib}^{b}$ is a skew-symmetric form of the angular velocity vector ${\tilde{\omega }}_{ib}^{b}$, and $T_{b}^{n}$ is the transformation matrix from body to navigation frame given by:

(6) $$T_{b}^{n}=\left[\begin{array}{ccc} C_{\theta }C_{\psi } & S_{\phi }S_{\theta }C_{\psi }-C_{\phi }S_{\psi } & C_{\phi }S_{\theta }C_{\psi }+S_{\phi }S_{\psi }\\ C_{\theta }S_{\psi } & S_{\phi }S_{\theta }S_{\psi }+C_{\theta }C_{\psi } & C_{\phi }S_{\theta }S_{\psi }-S_{\phi }C_{\psi }\\ -S_{\theta } & S_{\phi }C_{\theta } & C_{\phi }C_{\theta } \end{array}\right]$$

where $S_{x}$ is the sine of *x* and $C_{x}$ is the cosine of *x*.

### 2.2. Quadrotor Dead Reckoning

**Figure 2:**
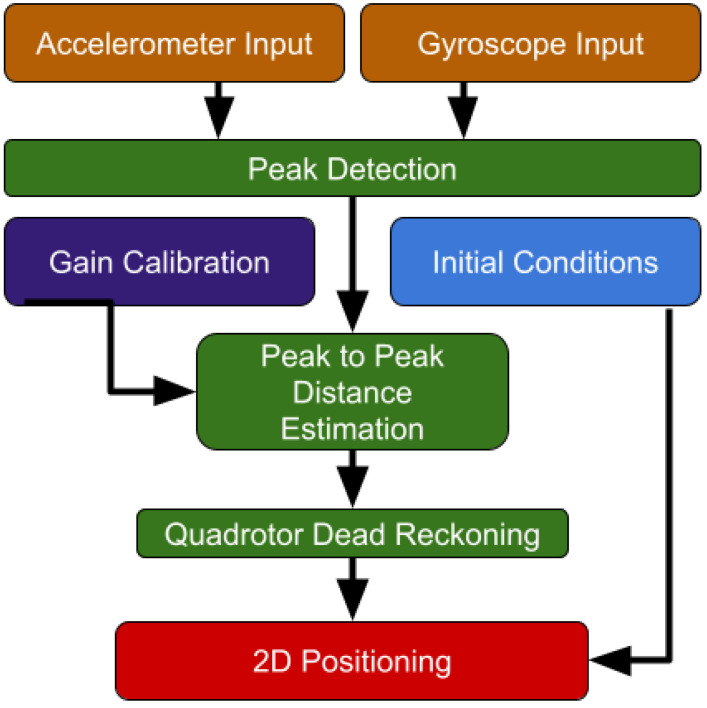
Block diagram of the quadrotor dead reckoning algorithm.

To cope with situations of quadrotors’ pure inertial navigation, the QDR approach, illustrated in Figure 2, was proposed [19]. The main idea was to fly the quadrotor in a periodic motion trajectory (instead of a straight line) to emulate a walking pedestrian, enabling the application of PDR approaches.

During the quadrotor motion, accelerometer readings are used to detect peaks, followed by a peak-to-peak (p2p) distance estimation using the Weinberg approach [40]. The attitude of the quadrotor is calculated in the same manner as in a traditional INS using (5). Instead of using (3), in situations of pure inertial navigation, given the quadrotor’s initial position $x_{k}$, $y_{k}$ at time *k*, the QDR approach uses the current p2p distance $d_{k}$ and heading $\psi_{k}$ to calculate the quadrotor’s horizontal position at time k+1 by:

(7) $$\begin{array}{ccc} x_{k+1} & = & x_{k}+d_{k}\cos\psi_{k} \end{array}$$

(8) $$\begin{array}{ccc} y_{k+1} & = & y_{k}+d_{k}\sin\psi_{k} \end{array}$$

The p2p distance is estimated using the Weinberg approach:

(9) $$d_{w}=G_{w}{\left(\max\left(f_{p2p}^{b}\right)-\min\left(f_{p2p}^{b}\right)\right)}^{1/4}$$

where $f_{p2p}^{b}$ is the set of the specific force magnitudes in the peak-to-peak duration, and $G_{w}$ is a precalibrated gain.

To calculate $G_{w}$ the quadrotor should be flown to a known distance with the required periodic motion. To maintain a high accuracy level, this procedure is repeated several times for each different periodic motion.

## 3. Quadnet1 Framework

### 3.1. Proposed Approach

In pure inertial navigation situations, the main challenge is to mitigate the navigation solution drift caused by the inertial sensors’ noisy measurements. As discussed in Section 2.2, the QDR approach offers a solution; however, it contains three drawbacks:

1. The required Weinberg gain in (9) requires precalibration and is very sensitive to the quadrotor periodic motion type.
2. QDR provides a position solution between two successive peaks only (p2p), where the time duration between the two peaks is several seconds.
3. The QDR approach does not provide the quadrotor altitude.

Thus, QDR gives a novel perspective for handling pure inertial situations and lays the foundations of such solution directions, yet it suffers from three drawbacks.

To circumvent QDR’s drawbacks, we propose QuadNet, a hybrid framework to estimate the quadrotor three-dimensional position vector at a user defined time rate. As a hybrid approach, QuadNet uses both neural network (NN) and model-based equations.

NN are employed as they are well-known in solving complex problems that require discovering hidden patterns in the data and/or a deep understanding of intricate relationships between a large number of interdependent variables. NN algorithms are able to learn hidden patterns from the data by themselves, combine them together, and build an efficient decision rule algorithm.

**Figure 3:**
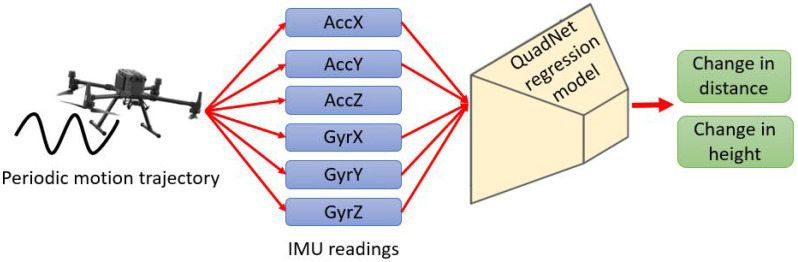
QuadNet’s end-to-end part regresses the change in distance and height using only the inertial sensor measurements.

As in QDR, QuadNet requires the quadrotor to be flown in a periodic motion. Data-driven approaches are utilized to estimate the quadrotor position vector. In this manner, raw accelerometer and gyroscope readings are plugged into a regression model in an end-to-end fashion, to regress the change distance and altitude of the quadrotor as presented in Figure 3.

In the model-based part of the QuadNet framework, gyroscope readings are introduced into (5) to update the transformation matrix and obtain the heading angle. Finally, the QuadNet equations of motion are:

(10) $$\begin{array}{ccc} x_{k+1} & = & x_{k}+{\Delta p}_{k}\cos\psi_{k} \end{array}$$

(11) $$\begin{array}{ccc} y_{k+1} & = & y_{k}+{\Delta p}_{k}\sin\psi_{k} \end{array}$$

(12) $$\begin{array}{ccc} z_{k+1} & = & z_{k}+\Delta h_{k} \end{array}$$

where ${\Delta p}_{k}$ is the regressed change in distance, $\Delta h_{k}$ is the regressed change in height, $\psi_{k}$ is the heading angle, and $x_{k},y_{k},z_{k}$ are the quadrotor position vector components.

The proposed hybrid framework does not require any prior gain calibration as in QDR. However, as a data-driven approach, a training process is required to obtain the NN architecture and hyper-parameters.

### 3.2. Quadnet Regression Model Architectures

Two different NN structures are considered for QuadNet’s regression model:

- **QuadNet1**: consisting of one-dimensional convolution neural networks (CNN) and fully connected layers;
- **QuadNet2**: consisting of one-dimensional CNN, long short-term memory (LSTM) neural networks, and fully connected layers.

Both structures are used to regress the change in distance and height. In the following sections, we elaborate on the NN structures.

#### 3.2.1. Quadnet 1

**Figure 4:**
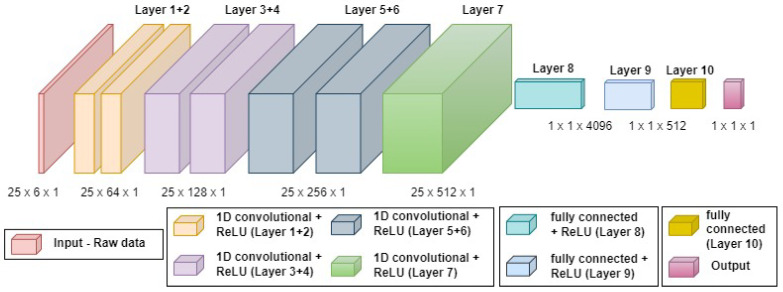
QuadNet1 architecture. QuadNet1 consists of 1D-CNN layers, used for feature extraction, and fully connected layers to output the change in distance or height.

Figure 4 presents the QuadNet1 architecture.

QuadNet1 is a deep neural network, which is a network with multiple hidden layers between the input and output layers. Denoting x as the input layer, the first hidden layer is defined by:

(13) $$h^{\left(1\right)}=g^{\left(1\right)}\left(W^{{\left(1\right)}^{T}}x+b^{\left(1\right)}\right)$$

where W is the weight matrix, b is the bias vector, and g is a nonlinear activation function. The weights and biases create a mapping between the neurons in the current layer and the neurons from the previous layer, and the activation function allows the model to predict a range of cases that are not linear in nature. Here, the commonly used activation function, the rectified linear unit (ReLU) [41], is employed:

(14) g(z)=max{0,z}

where z is the input to the activation function.

The *i*-th layer is defined by:

(15) $$h^{\left(i\right)}=g^{\left(i\right)}\left(W^{{\left(i\right)}^{T}}h^{\left(i-1\right)}+b^{\left(i\right)}\right)$$

where $h^{\left(i-1\right)}$ is the output of the previous layer.

QuadNet1 has seven one-dimensional CNNs and three fully connected layers (13)–(15) that are used to output a single value, which is the change in distance or height. CNN is employed in this architecture as it has had groundbreaking results over the past decade in a variety of fields. One of CNN’s biggest advantages is that it significantly reduces the number of parameters and thus allows us to build larger networks.

The convolution is defined by:

(16) $$\left(x*W\right)\left(t\right):=\int_{-\infty }^{\infty }x\left(\tau \right)W\left(t-\tau \right)d\tau$$

where x is the input, W is the filter matrix, and (∗) is the convolution operator. The filter matrix is referred to as the weights matrix, which is updated during the training process.

The input to QuadNet1 is the raw accelerometer and gyroscope readings, and the output is the change in distance or height.

#### 3.2.2. Quadnet 2

**Figure 5:**
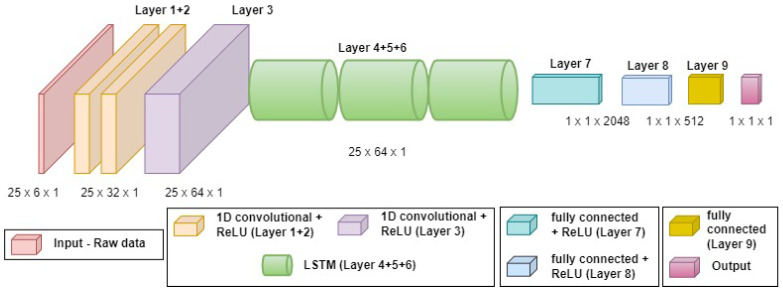
QuadNet2 architecture. A combination of CNN network for feature extraction and LSTM for regression.

QuadNet2 structure, presented in Figure 5, is a combination of three CNN layers for feature extraction, three LSTM layers, and three fully connected layers.

The LSTM network processes a sequence of input and target pairs. For each pair, the LSTM network takes the new input and produces an estimate for the target given all the previous inputs [42]. The LSTM has the ability to remove or add information to the cell state, carefully regulated by structures called gates. Gates are a way to optionally let information through. They are composed of a sigmoid neural net layer and a pointwise multiplication operation:

1. **Forget Gate:** Decides what information is thrown away from the cell state:
  (17) $$f_{t}=\sigma \left(W_{f}\left[h_{t-1},x_{t}\right]+b_{f}\right)$$
  where $f_{t}$ is the forget gate’s activation vector, σ is the sigmoid function, $W_{f}$ is the weights matrix, $h_{t-1}$ is the hidden state vector of the previous layer, $x_{t}$ is the input vector to the LSTM unit, and $b_{f}$ is the bias vector.
2. **Input Gate:** Decides which values are updated by:
  (18) $$i_{t}=\sigma \left(W_{i}\left[h_{t-1},x_{t}\right]+b_{i}\right)$$
  where $i_{t}$ is the update gate’s activation vector.
3. **Cell Input State:** Creates a vector of new candidate values that could be added to the state. Together with (18), it is used to create an update for the state:
  (19) $${\tilde{C}}_{t}=\tanh\left(W_{C}\left[h_{t-1},x_{t}\right]+b_{C}\right)$$
  where ${\tilde{C}}_{t}$ is the cell input activation vector.
4. **Cell State:** The old state is multiplied by the forget gate’s activation function, and the input gate’s activation vector is multiplied by the cell input activation vector to obtain the updated cell state:
  (20) $$C_{t}=f_{t}\cdot C_{t-1}+i_{t}\cdot {\tilde{C}}_{t}$$
5. **Output Gate and Hidden State**: The output is based on the previous layer’s hidden state, while the hidden state is based on the cell state as described by:
  (21) $$\begin{array}{ccc} o_{t} & = & \sigma (W_{o}\left[h_{t-1},x_{t}\right]+b_{o}) \end{array}$$
  (22) $$\begin{array}{ccc} h_{t} & = & o_{t}\cdot \tanh\left(C_{t}\right) \end{array}$$
  where $o_{t}$ is the output gate’s activation vector, and $h_{t}$ is the hidden state vector.

QuadNet2 has the same input and output as QuadNet1. The main difference is that after the inertial sensor raw data goes through the CNN layers for feature extraction, they are fed through LSTM layers for the regression process.

### 3.3. Loss Function

In both QuadNet1 and QuadNet2, the same loss function is used. The result of the output layer is compared to the ground-truth (GT) labels by using a loss function. By minimizing it, an accurate network approximation is achieved. The minimization of the loss function is performed by the back propogation process, where the loss function is derived and the weights are updated accordingly. Those two processes continue until a global minimum is reached. For the problem at hand, the goal is to regress the change in distance or height.

As a consequence, we want large errors to be penalized more significantly (quadratically) than small errors, enabling the network to estimate the desired output in a variety of conditions. To that end, the mean squared error (MSE) Loss function is employed:

(23) $$\mathrm{MSE}\;\mathrm{Loss}\left(y_{i},\hat{y_{i}}\right)=\frac{1}{N}\sum_{i=1}^{N}{\left(y_{i}-\hat{y_{i}}\right)}^{2}$$

where *N* is the number of examples, $y_{i}$ is the GT label observed at *i*, and $\hat{y_{i}}$ is the estimated value observed at *i*.

## 4. Data Collection and Preprocessing

To evaluate QuadNet’s performance relative to the QDR approach, the same dataset as in [19] was used. This dataset was recently published in the autonomous platforms inertial dataset [43] and is available at https://github.com/ansfl/Navigation-Data-Project/, accessed on 22 December 2020.

**Figure 6:**
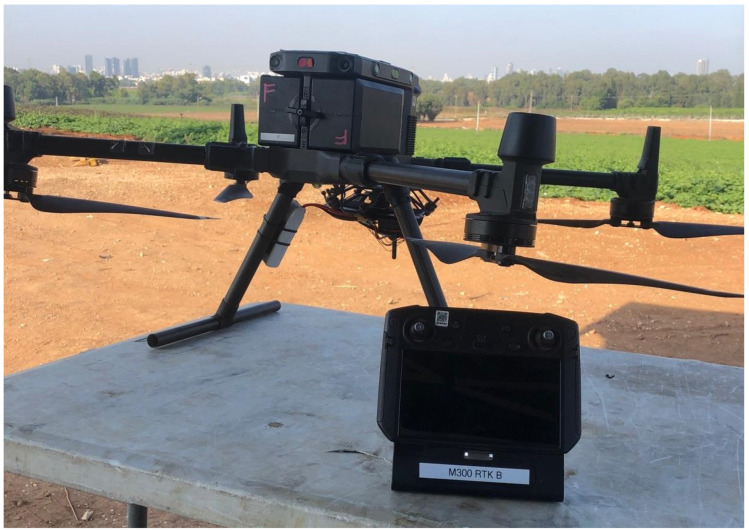
DJI Matrice 300 RTK used in the field experiments.

DJI’s Matrice 300 RTK quadrotor, as shown in Figure 6, was used in the experiments. The experiments were performed in an outdoor environment for GNSS measurements availability. A Pixhawk Cube flight controller was mounted on the quadrotor landing gear to record its three IMU sets during flight. Note that out of the three IMUs, only two sets are mechanically vibration-isolated; therefore, the third IMU readings are not used in this work.

In addition, an RTK GNSS receiver was installed next the Pixhawk Cube to obtain the GT of the trajectory. The sampling rate of Pixhawk Cube IMU is 25 Hz, and the sampling rate of the RTK GPS is 10 Hz.

The quadrotor was flown manually by an experienced and licensed pilot. The travelled distance of the trajectory we used in the experiment was about 100 m, and the altitude was set to 20 m. The quadrotor was flown in a straight line and in a periodic motion back and forth to the same starting point. Due to disturbances caused by manual flight and strong winds, the altitude of the quadrotor was not kept constant during the straight flight, and the periodic motion was not smooth.

### 4.1. Dataset

A total of 15.6 min of recording data consisting of GNSS and IMU measurements was collected during 15 trajectories.

Those include 14 trajectories with periodic motion and 1 straight line trajectory. The former are used for comparison to the classical pure inertial navigation solution.

Focusing on the periodic motion trajectories, 12 trajectories with a total flight time of 14.2 min were used for training and 2 trajectories with a total flight time of 53 s were used for testing. For both train and test datasets, the accelerometer and gyroscope readings were used as input. As two sets of IMU are used, the train dataset contains 67,800 samples over 6 inertial sensors, i.e., a total of 406,800 reading samples.

Training a neural network requires a large amount of data, which means a large amount of recordings is needed. The number of recorded samples we have is considered small; therefore, data augmentation was applied to achieve more examples using the same recorded trajectories.

The number of total samples we have in our dataset is calculated as:

(24) Samples=SamplingRate·TotalRecordingTime

The number of samples inserted as input to the neural network is of our choosing and is called the window size. The number of examples we can create for our dataset depends on the number of samples, window size, and the stride we choose, as described in:

(25) $$Examples=\frac{Samples-Window\;Size}{Stride}+1$$

where stride is the number of sample shifts over the input matrix. For example, when the stride is equal to one, then we move the window one sample at a time.

Since our sampling rate is 25 Hz, by choosing a window size of 25 samples and a stride of 25, we obtain a number of examples that is equal to the recorded time of the trajectory. However, by choosing a stride value lower than 25 samples, we create a bigger number of examples while simultaneously creating an overlap between the data that is described as:

(26) Overlap=SamplingRate−Stride.

For example, if we look at a range of [1–50], by using a stride of 25, we obtain 2 examples in the ranges of [1–25] and [26–50]. However, by using a stride of five, we obtain six examples in the ranges of [1–25], [5–30], [10–35], and up until [25–50].

For our analysis, we choose a stride of 5; hence, we have an overlap of 20 samples, and by that we obtain around 4200 examples, i.e., the size of our training dataset is [4200,6,25]. For the test dataset, a stride of 25 samples is chosen to avoid overlap. In the test dataset, the time duration of the first trajectory is 23 s, and the second trajectory duration is 30 s, resulting in dimensions of [23,6,25] and [30,6,25].

For the training process, a batch size of 32, a constant learning rate of 0.0001, and the ADAM optimizer were used. The QuadNet networks were trained on the Google Colab GPU.

## 5. Analysis and Results

### 5.1. Performance Measure

The root mean square error (*RMSE*) metric and the distance error at the end of the trajectory were chosen as the performance measure, where the *RMSE* was used for both the distance and height.

The *RMSE* is defined by:

(27) $$\mathrm{RMSE}\left(x_{i},\hat{x_{i}}\right)=\sqrt{\frac{\sum_{i=1}^{N}{\left(x_{i}-\hat{x_{i}}\right)}^{2}}{N}}$$

where *N* is the number of samples, $x_{i}$ is the GT distance/height observed at time *i*, and $\hat{x_{i}}$ is the estimated position/height observed at time *i*.

### 5.2. Baseline Architecture Evaluation: Distance

**Figure 7:**
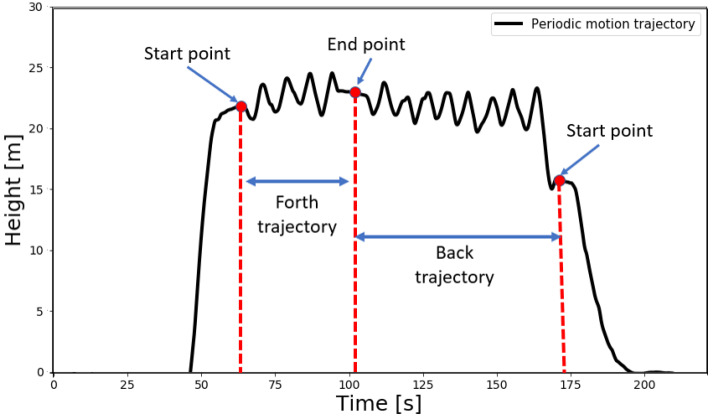
Quadrotor height variation during the periodic motion trajectories. Height was obtained using RTK-GPS measurements.

Equations (3)–(5) were used to calculate the pure inertial navigation solution for the straight line trajectory. The test dataset containing the periodic motion trajectories was used to evaluate the QDR and QuadNet approaches. Each of those trajectories was recorded in a back-to-forth manner; that is, flying from a start point to the end point and back to the start point as shown in Figure 7. Note that the periodic motion is not repeatable between peaks due to pilot flight errors and winds that were present during the flights. The quadrotor height was obtained using RTK-GPS measurements.

Prior to using the QDR approach, the approach gain is calculated. To that end, the train dataset was employed to obtain a QDR gain of 14.07 using (9). This gain is used by applying the QDR position calculations (7)–(8).

Using the same train dataset (containing the two IMUs readings), both QuadNet architectures (Section 3.2) were trained. The evaluation of QDR and QuadNet was made on the two trajectories in the test dataset, where for each trajectory two different IMUs were examined.

For all three approaches—INS, QDR, and QuadNet—the heading angle is obtained from (5). The same procedure was repeated for each of the two IMUs.

Note that the INS approach and QuadNet frameworks are used to estimate both the change in distance and height of the quadrotor, while the QDR approach is used for distance estimation only.

Table 1 presents the results obtained on the test dataset using the three approaches: INS, QDR, and QuadNet, for distance estimation using the first IMU.

Both QuadNet architectures outperformed the INS and QDR approaches for the position at the end of the trajectory and RMSE. INS results for the straight line trajectory showed an error of 1.4 times the traveled distance, with an RMSE of 41.8 m. In the QDR approach, the error at the end of the trajectory was less than 2.2% with an RMSE less than 5 m. In QuadNet2, the error was less than 2% and RMSE of 2.1 m, reducing more than half of the QDR’s RMSE. QuadNet1 achieved the best results as its error was less than 0.1% and RMSE less than 1.8 m for both trajectories.

**Figure 8:**
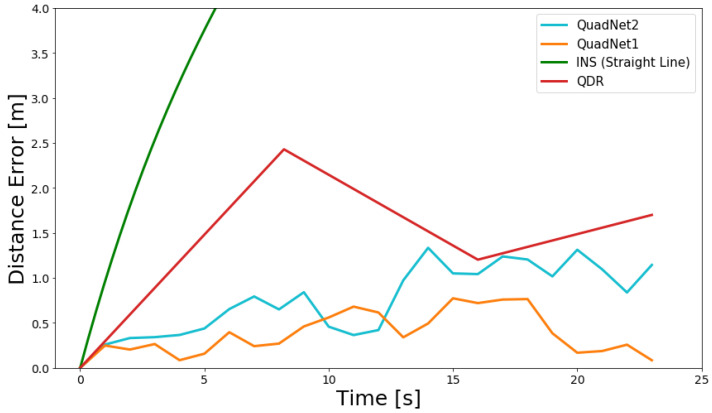
INS, QDR, and QuadNet architectures distance error throughout the periodic motion trajectory #1 using IMU #1.

**Figure 9:**
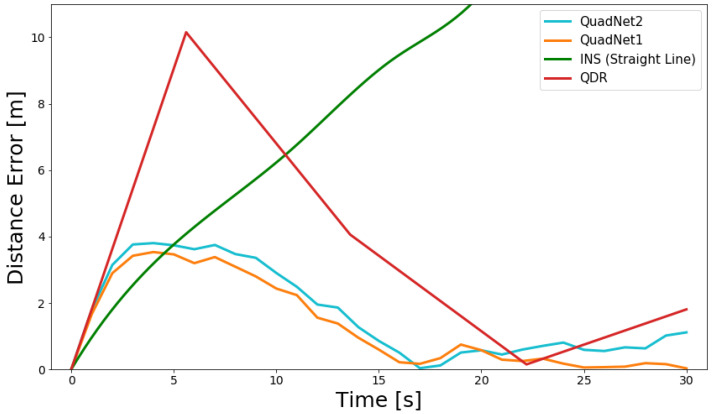
INS, QDR, and QuadNet architectures distance error throughout the periodic motion trajectory #2 using IMU #1.

The QuadNet frameworks also maintain better tracking of the actual trajectory, as the QDR approach updates the distance only between two peaks, while in QuadNet, the distance update occurs every 1 s (25 IMU samples). To better illustrate that, Figure 8 and Figure 9 present the distance error throughout the two test trajectories.

The pure inertial INS trajectories rapidly diverge. The QDR approach provides an estimation of the change in distance approximately every 7 s.

The same procedure was repeated for IMU #2, and the results are given in Table 2.

Once again, both QuadNet architectures show better results. INS results for the straight line trajectory showed an error of 4.6 times the traveled distance, with an RMSE of 128.2 m. In the QDR approach, the error at the end of the trajectory was less than 9% with an RMSE less than 10 m. In QuadNet2, the error was less than 8%, and the RMSE was less than 4 m. The QuadNet1 error was less than 5% and the RMSE was less than 3 m for both trajectories.

### 5.3. Baseline Architecture Evaluation: Height

The QDR approach follows PDR guidelines and therefore allows only the estimation of the change in distance. Thus, the QDR approach is not considered in this section.

Following the same procedure as in the previous section, Table 3 shows the height RMSE of the traditional INS approach and both QuadNet architectures using IMU #1 readings.

It is clearly seen that QuadNet approaches outperformed the traditional INS. QuadNet2 showed an RMSE of less than 1.8 m, while the INS’s RMSE was more than 14.7 m. As in the distance estimation, QuadNet1 achieves the superior results with an RMSE less than 1 m, improving QuadNet2 by 61.5% in the first trajectory and by 61.1% in the second trajectory.

The same procedure was repeated for IMU #2, and the results are given in Table 4.

The same behavior is seen in the second trajectory. Both QuadNet architectures greatly improved the INS performance, and QuadNet1 obtained the best result. This time, the amount of improvement relative to QuadNet2 was 10% and 34% for trajectories 1 and 2, respectively.

**Figure 10:**
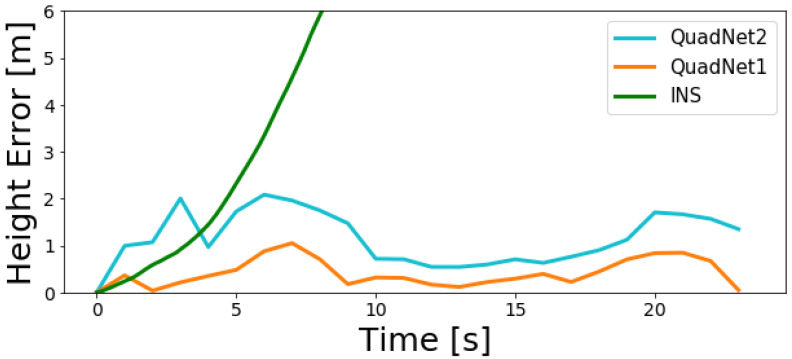
INS, QuadNet2, and QuadNet1 calculated height error throughout the trajectory for the first test dataset.

**Figure 11:**
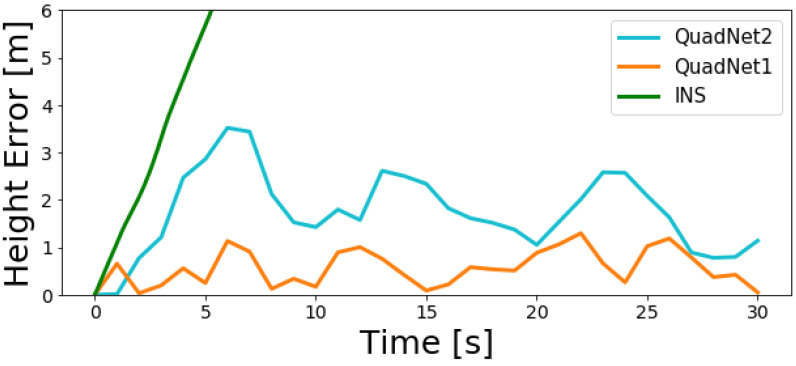
INS, QuadNet2, and QuadNet1 calculated height error throughout the trajectory for the second test dataset.

Figure 10 and Figure 11 show the INS and both deep learning frameworks calculated height error throughout the two trajectories.

As observed in the figures, the INS solution quickly diverges while both QuadNet architectures achieve a bounded error. Along the entire trajectories, a height error of less than 4 m was obtained for QuadNet2 and less than 1.5 m for QuadNet1. That is, QuadNet2 achieves an error that is more than twice the error of QuadNet1. In addition, it is easily seen that the trajectory tracking ability, as well as the overall accuracy of the proposed approach, is much better compared to the INS in situations of pure inertial navigation.

### 5.4. Influence of Window Size

In both distance and height evaluations, QuadNet1 obtained the best performance using the baseline network parameters defined in Section 4.1. To seek better performance, the influence of window size was examined using the QuadNet1 architecture. The baseline window size is 1s, corresponding to 25 samples as the IMUs operate at 25 Hz. For our analysis, window sizes of 15 and 50 samples are also examined.

Table 5 shows the distance and height RMSE for the three window sizes using QuadNet1 architecture and IMU #1 measurements.

The 25 sample window size showed the best results for both distance and height RMSE while the 15-sample window size obtained the worst performance. This is attributed to the fact that a 15-sample window size is too small for the network to learn the data given the quadrotor dynamics. Moreover, by using a 50-sample window size, the distance and height RMSE of trajectory #1 worsen by 16%. Yet, regardless of the window size, the INS errors (distance RMSE of 143 m, as shown in Table 1, and height RMSE of 20.7 m, as shown in Table 5) greatly improve. Thus, the window size hyper-parameter should be tuned as a function of the IMU measurement rate and the quadrotor dynamics to minimize the RMSE.

### 5.5. Influence of Input Size

Until now, the input of the QuadNet networks has a dimension of six and includes the three gyroscope and three accelerometer measurements. Here, we examine two more possibilities for the input dimension:

1. **Six (baseline)**: Three gyroscope and three accelerometer measurements; i.e., the specific force and angular velocity vectors.
2. **Three**: Three accelerometer measurements; that is, only the specific force vector.
3. **Two**: The specific force and angular velocity magnitudes.

Table 6 shows the distance and height RMSE for the QuadNet1 architecture using IMU #1 measurements.

In general, it is expected that by inserting wider data to the network it will achieve better performance, particularly because both accelerometers and gyroscopes are used to determine the position of the quadrotor using INS equations. Therefore, it is not surprising that when the input includes both accelerometer and gyroscopes measurements, QuadNet obtains the best performance.

Nevertheless, all input types greatly improve the pure inertial INS solution (143 m, as shown in Table 1). As a result, with a cost of reduced performance compared to the baseline input, only accelerometers can be used to greatly reduce the INS solution. This could be very beneficial when strict weight or power constraints are met, such as in micro-drones.

## 6. Conclusions

QuadNet, a hybrid framework for quadrotor dead reckoning using only inertial sensors was proposed to estimate the quadrotor’s position vector. To that end, the quadrotor is required to be flown in a periodic motion trajectory. QuadNet was destined to improve the QDR approach and improve the navigation accuracy in situations of pure inertial navigation. Specifically, QDR depends on a precalibrated gain, provides only peak-to-peak distance estimation, and does not estimate the quadrotor altitude. The proposed QuadNet framework does not require any gain and provides the change in distance and altitude of the quadrotor in any required time duration.

Two QuadNet architectures—QuadNet1 and QuadNet2—were examined for the regression task. The first comprises CNN layers, while the second is a combination of CNN layers and LSTM. A comprehensive comparison between the two frameworks, the QDR approach, and traditional INS on the same dataset was conducted. The dataset was collected using DJI Matrice 300 RTK and includes two sets of inertial sensors and RTK GNSS GT solution.

Among all approaches, the QuadNet1 architecture (CNN only) achieved superior results with an average error less than 0.5% of the traveled distance, a distance RMSE of 1.5 m, and an altitude RMSE less than 1 m. Compared to QuadNet2, QuadNet1’s performance is better by 1.5 times in the distance RMSE and 1.6 times in altitude RMSE.

We also showed that using only accelerometer readings as the input to the NN greatly improves the standalone INS solution. As a consequence, for micro-drones or when limitations on the power/cost/computational load exist, QuadNet can be applied using only accelerometers.

As for QDR, QuadNet requires to be flown in a periodic motion trajectory, and so a trade-off is considered between reduced energy consumption in straight line trajectories to a more accurate navigation solution in periodic motion trajectories. Considering that short time periods are most common situations for pure inertial navigation, in most scenarios the added energy consumption can be ignored.

## Figures and Tables

**Table 1:** INS, QDR, and QuadNet distance estimation errors for IMU #1.

Trajectory	Actual Distance [m]	Approach	Dist. Error [m]	RMSE [m]
Straight line	101.1	INS	143	41.8
		QDR	1.7	1.6
Periodic Motion #1	80.2	QuadNet1	0.08	0.45
		QuadNet2	1.15	0.85
		QDR	1.8	4.95
Periodic Motion #2	95.9	QuadNet1	0.02	1.8
		QuadNet2	1.1	2.1

**Table 2:** INS, QDR, and QuadNet distance estimation errors for IMU #2.

Trajectory	Actual Distance [m]	Approach	Dist. Error [m]	RMSE [m]
Straight line	101.1	INS	459.7	128.2
		QDR	6.9	6.6
Periodic Motion #1	80.2	QuadNet1	2.1	1
		QuadNet2	3.0	2.3
		QDR	6.9	9.2
Periodic Motion #2	95.9	QuadNet1	4.3	2.8
		QuadNet2	6.8	3.9

**Table 3:** INS and QuadNet height RMSE using IMU #1 readings.

Trajectory	Approach	RMSE [m]
	INS	20.7
Periodic Motion #1	QuadNet1	0.5
	QuadNet2	1.3
	INS	14.7
Periodic Motion #2	QuadNet1	0.7
	QuadNet2	1.8

**Table 4:** INS and QuadNet height RMSE using IMU #2 readings.

Trajectory	Approach	RMSE [m]
	INS	78.7
Periodic Motion #1	QuadNet1	1.3
	QuadNet2	1.45
	INS	96.1
Periodic Motion #2	QuadNet1	1.25
	QuadNet2	1.9

**Table 5:** Influence of window size on QuadNet1 performance using IMU #1 measurements.

Trajectory	Window Size	Distance RMSE	Height RMSE
	[Samples]	[m]	[m]
	15	1.4	1.5
Periodic Motion #1	25	0.5	0.5
	50	0.6	0.6
	15	2.6	1.7
Periodic Motion #2	25	1.8	0.7
	50	1.9	1.1

**Table 6:** Influence of input size on QuadNet1 performance using IMU #1 measurements.

Trajectory	Input Size	Distance RMSE	Height RMSE
	[Samples]	[m]	[m]
	2	13.8	2.5
Periodic Motion #1	3	3.35	0.7
	6	0.45	0.5
	2	16.75	1.6
Periodic Motion #2	3	4.15	1.05
	6	1.8	0.7

## Data Availability

Dataset is available at 22 December 2020 https://github.com/ansfl/Navigation-Data-Project/.
